# CMR-derived ECVs vary with myocardial region and associate with the regional wall thickness

**DOI:** 10.1038/s41598-020-78043-5

**Published:** 2020-12-01

**Authors:** Mao-Yuan Su, Kuei-Yuan Hou, Ming-Hung Liu, Tien-Min Lin, Jyh-Ming Jimmy Juang, Lian-Yu Lin, Cho-Kai Wu, Hsi-Yu Yu, Shun-Chung Yang, Yu-Sen Huang, Emi Niisato, Yeun-Chung Chang

**Affiliations:** 1 Department of Medical Imaging, National Taiwan University Hospital, Taipei, 10002 Taiwan, ROC; 2 Department of Medical Imaging, Cathay General Hospital, Taipei, 10630 Taiwan, ROC; 3 Department of Biomedical Imaging and Radiological Sciences, National Yang-Ming University, Taipei, 11221 Taiwan, ROC; 4 Cardiovascular Center and Division of Cardiology, Department of Internal Medicine, National Taiwan University Hospital, Taipei, 10002 Taiwan, ROC; 5 Department of Surgery, National Taiwan University Hospital, Taipei, 10002 Taiwan; 6 Siemens Healthcare Limited, Taipei, 11503 Taiwan, ROC; 7 Department of Medical Imaging and Radiological Technology, Yuanpei University of Medical Technology, Hsinchu, Taiwan, ROC

**Keywords:** Biophysics, Biotechnology, Cardiology

## Abstract

This study was designed to identify whether the position and size of the region of interest (ROI) influence extracellular volume fraction (ECV) measurements. Patients with localized (n = 203) or infiltrative (n = 215) cardiomyopathies and 36 normal controls were enrolled in this study. ECV measurements at 4 different regions, including the anterior, septal, posterior and lateral wall regions, were measured, and all groups were compared. Regional ECV was correlated with the corresponding regional wall thickness. The diagnostic power to differentiate the myocardial abnormalities was evaluated for each myocardial region. ECVs measured using five different ROI sizes within each myocardial region were compared. Our results showed that ECVs varied among the myocardial regions, and this variation was significantly associated with regional wall thicknesses. For the detection of myocardial abnormalities, regional ECV revealed similar results as ECV derived from the whole region except for the anterior region. No significant difference was found in the ECVs measured using the five different ROI sizes. In conclusion, CMR-derived ECVs vary with myocardial region, and this variation is significantly associated with the regional wall thickness. In contrast, the measured size of the ROI has less of an effect on the ECV.

## Introduction

Cardiovascular magnetic resonance (CMR) has been widely used to quantify myocardial interstitial matrix by calculating the extracellular volume fraction (ECV) of the myocardium^1–4^. Quantitative analysis of myocardial ECV can be performed by measuring T1 in blood and myocardium before and after administration of contrast medium. T1 measurement is most often performed by drawing a region of interest (ROI) in the central area of the left ventricle (LV) cavity and the septal myocardium on pixel-wise T1 mapping ^5–9^. The position of ROIs is determined first, and the average T1 values within the ROIs are then computed. For the assessment of diffuse myocardial abnormality, the position of the ROI can be drawn within the myocardium, except for the regions with enhancement shown on late gadolinium enhancement (LGE) imaging. This method assumes that the diffuse myocardial abnormality is distributed homogeneously (uniform ECV) within the noninfarcted regions. However, the spatial variation of diffuse myocardial abnormality is diverse and depends on the various cardiomyopathies ^10,11^. Therefore, the ECV measurement could be affected by the position of the ROI if significant regional variation in the interstitial matrix exists. Furthermore, the size of the ROI is arbitrarily drawn, and whether it is a confounder in the quantification of the ECV is unknown. To measure subtle changes in the ECV expected for diffuse myocardial abnormality, it is essential to circumvent factors that confound the ECV measurement using the ROI-based method.

In this study, we evaluated the ECV from different myocardial regions and compared them in both the patient and control groups. ECV values measured from different sizes of ROI within the same region were also compared. Our goal was to identify whether the regional position and the measured size of ROIs affect the quantification of the ECV.

## Results

### Patient characteristics

The demographics of the study population are summarized in Table 1. Compared with the control group, the patient groups were older and included more males; however, body surface areas were similar. There were significant differences in LV function and mass at end-diastole between the patient and control groups. In the patient group, ten different types of cardiomyopathies were included for further analysis. The homogeneous disease (H.D.) group (n = 215) included cardiac amyloidosis (n = 15), arrhythmogenic right ventricular cardiomyopathy (n = 47), Brugada syndrome (n = 64), dilated cardiomyopathy (n = 37), Fabry disease (n = 24) and heart failure with preserved ejection fraction (n = 28). The regional disease (R.D.) group (n = 203) included hypertensive cardiac disease (n = 81), hypertrophic cardiomyopathy (n = 87), ischemic cardiomyopathy (n = 21) and myocarditis (n = 14).

**Table 1:** Characteristics of the patients and normal controls.

	R.D. group (n = 203)	H.D. group (n = 215)	Ctr. Group (n = 36)
**Demographic**			
Age, years (range)	61 (18–98)	49 (10–78)^†^	30 (21–58)^†^
Male sex, (%)	127 (63)	148 (69)	12 (40)^†^
BSA, m^2^	1.76 ± 0.18	1.75 ± 0.22	1.68 ± .68
Hct, (%)	42.5 ± 4.7	42.2 ± 4.5	42.9 ± 2.8
Time interval between the date of Hct and CMR, days (IQR)	130 (1–72)	93 (1–39)	0
Amyloidosis, (%)		15 (7)	
ARVC, (%)		47 (22)	
BrS (%)		64 (30)	
DCM, (%)		37 (17)	
Fabry, (%)		24 (11)	
HTC, (%)	81 (40)		
HCM, (%)	87 (43)		
HFpEF, (%)	28 (14)		
ICM, (%)	21 (10)		
Myocarditis, (%)	14 (7)		
**LV function by CMR**			
LVEDV_i_, ml/m^2^	54.4 ± 12.9	70.6 ± 32.5^†^	64.3 ± 12.3^†^
LVESV_i_, ml/m^2^	11.7 ± 8.97	25.9 ± 28.8^†^	18.7 ± 6.3^†^
EF, %	79.9 ± 9.9	69.4 ± 15.8^†^	72.2 ± 5.1^†^
PER, s^−1^	− 4.6 ± 1.19	− 3.48 ± 1.19^†^	− 3.49 ± 0.79^†^
PFR, s^−1^	4.02 ± 1.59	3.89 ± 1.66	5.52 ± 1.30*^†^
LVM_i_, g/m^2^	152 ± 66	121 ± 59^†^	88 ± 26*^†^

Values are mean ± SD unless stated.

*R.D.* regional disease; *H.D.* homogeneous disease; *Ctr.* control; *BSA* body surface area; *Hct* hematocrit; *IQR* interquartile range; *ARVC* arrhythmogenic right ventricular cardiomyopathy; *BrS* Brugada syndrome; *DCM* dilated cardiomyopathy; *Fabry* Fabry disease; *HTC* hypertensive cardiac disease; *HCM* hypertrophic cardiomyopathy; *HFpEF* heart failure with preserved ejection fraction; *ICM* ischemic cardiomyopathy; *LVEDV*_*i*_ left ventricular end-diastolic volume indexed; *LVESV*_*i*_ left ventricular end-systolic volume indexed; *EF* left ventricular ejection fraction; *PER* peak ejection rate; *PFR* peak filling rate; *LVM*_*i*_ left ventricular mass indexed at end-diastole.

*p < 0.05 for compared with R.D. group.

^†^p < 0.05 for compared with H.D. group.

### Group and subgroup regional comparisons in ECV

**Figure 1:**
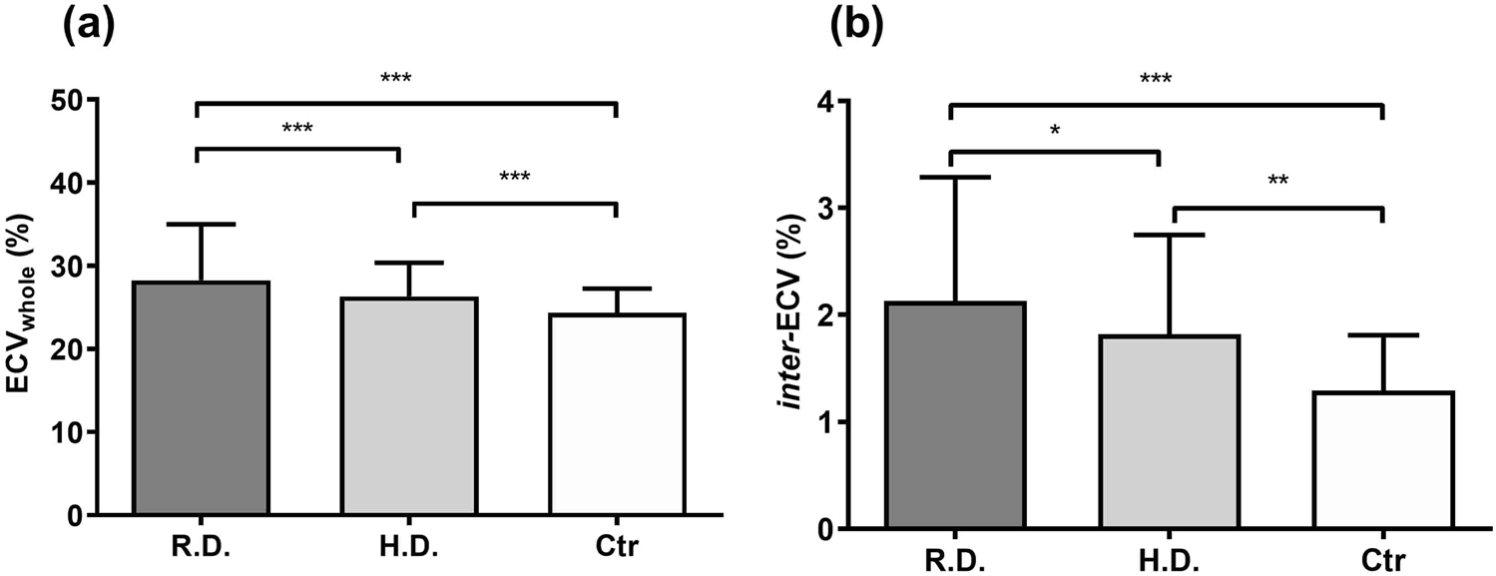
Group comparisons of the whole regional extracellular volume fraction (ECV) (**a**) and the interregional variation of ECV among the groups (**b**). *ECV*_*whole*_ the whole regional ECV; *inter-ECV* the interregional variation of ECV; *R.D.* regional disease; *H.D.* homogeneous disease; *Ctr.* control.

**Figure 2:**
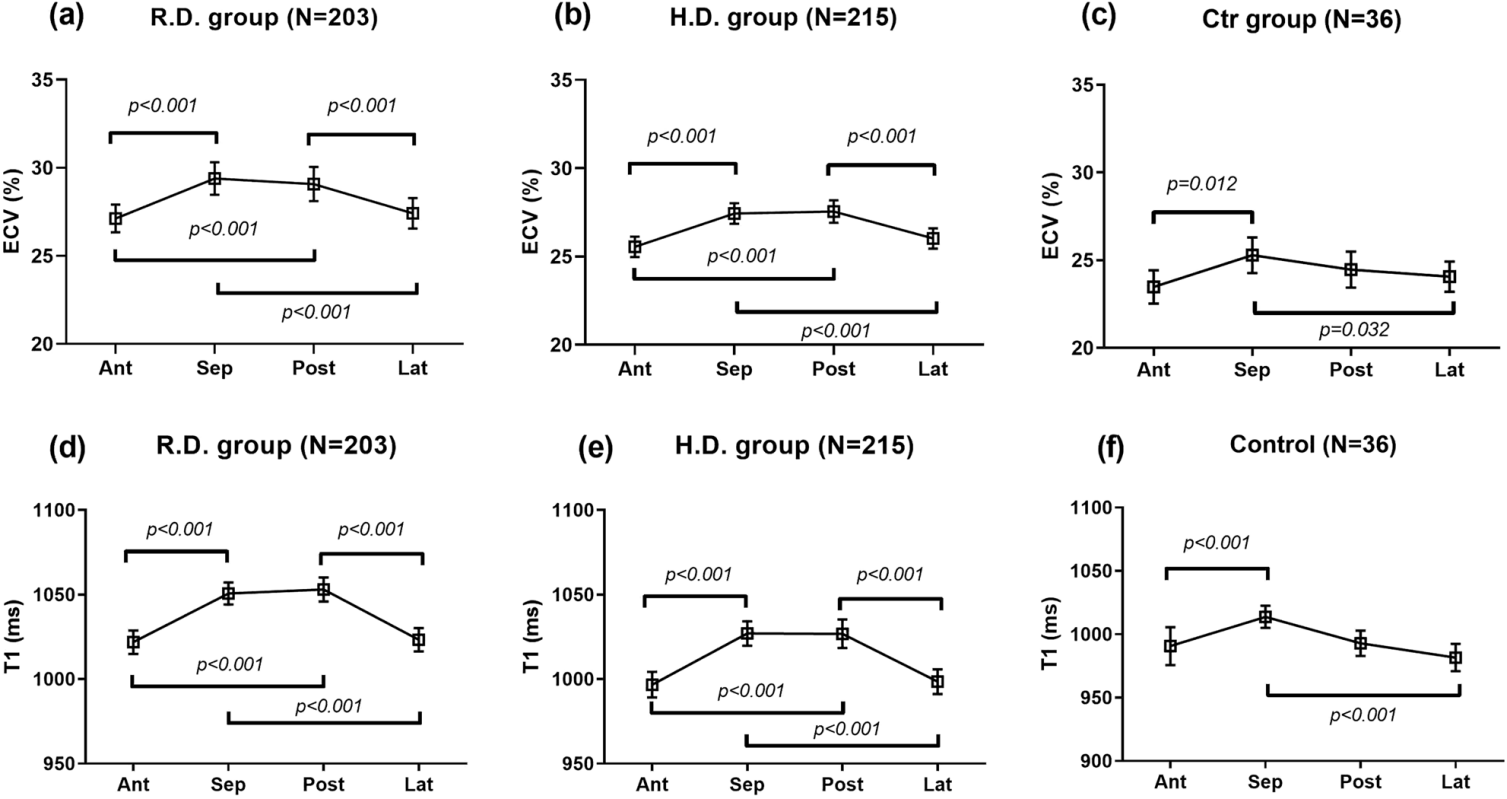
Extracellular volume fraction (ECV) and native T1 measured from four different regions in the regional disease (R.D.), homogeneous disease (H.D.), and controls (Ctr) groups. The data presented are the means and 95% confidential intervals.

For the group comparison, the R.D. group had a significantly higher ECV_whole_ compared to that in H.D. group (28.3 ± 6.7% vs. 26.3 ± 4.0%, p < 0.001) and in the control group (28.3 ± 6.7% vs. 22.6 ± 1.6%, p < 0.001). The H.D. group also had significantly higher ECV_whole_ compared with those of the control group (26.3 ± 4.0% vs. 22.6 ± 1.6%, p < 0.001) (Fig. 1a). The regional ECV (ECV_reg_) measurements from the four different regions in each group are listed in Table 2. For regional comparisons, the septal region had the highest ECV compared with the other three regions in all the groups. In the R.D. group, four significant ECV_reg_ differences were found. These were between the septal and anterior regions (29.4 ± 6.89% vs. 27.1 ± 5.93%, p < 0.001), the septal and lateral regions (29.4 ± 6.89% vs. 27.4 ± 6.48, p < 0.001), the posterior and later regions (29.1 ± 7.27% vs. 27.4 ± 6.48%, p < 0.001), and the posterior and anterior regions (29.1 ± 7.27% vs. 27.1 ± 5.93%, p < 0.001) (Fig. 2a). Similar ECV_reg_ differences were found in the H.D. group. These were between the septal and anterior regions (27.1 ± 3.88% vs. 25.2 ± 3.76%, p < 0.001), the septal and lateral regions (27.1 ± 3.88% vs. 25.7 ± 3.89%, p < 0.001), the posterior and later regions (27.2 ± 4.26% vs. 25.7 ± 3.89%, p < 0.001), and the posterior and anterior regions (27.2 ± 4.26% vs. 25.2 ± 3.76%, p < 0.001) (Fig. 2b). In the control group, two significant regional differences in the ECV_reg_ were found: between the septal and anterior regions (25.3 ± 3.01% vs. 23.5 ± 2.80%, p = 0.012) and the septal and lateral regions (25.3 ± 3.01% vs. 24.1 ± 2.55%, p = 0.032) (Fig. 2c). These interregional variation patterns were consistent with the native T1 in all groups (Fig. 2d–f). In contrast, no significant interregional variation was found in postcontrast T1 in each group.

**Table 2:** T1 and extracellular volume fraction (ECV) measurements of each myocardial region in the patient and control groups.

	Regions	R.D. group (n = 203)	H.D. group (n = 215)	Ctr. Group (n = 36)
Native T1 (ms)	Anterior	1028 ± 74.5	997 ± 53.9*	991 ± 44.1*
	Septal	1056 ± 71.5	1027 ± 51.4*	1014 ± 25.9*
	Posterior	1056 ± 78.5	1027 ± 60.4*	993 ± 29.5*
	Lateral	1028 ± 74.1	999 ± 52.8*	982 ± 31.9*
Postcontrast T1 (ms)	Anterior	576 ± 60.5	585 ± 53.9	514 ± 49.4*^†^
	Septal	565 ± 61.2	577 ± 51.8	508 ± 50.6*^†^
	Posterior	568 ± 65.4	563 ± 55.4	514 ± 53.6*^†^
	Lateral	574 ± 60.7	581 ± 53.2	516 ± 44.9*^†^
ECV (%)	Anterior	27.1 ± 5.93	25.2 ± 3.76*	23.5 ± 2.80*^†^
	Septal	29.4 ± 6.89	27.1 ± 3.88*	25.3 ± 3.01*^†^
	Posterior	29.1 ± 7.27	27.2 ± 4.26*	24.5 ± 3.03*^†^
	Lateral	27.4 ± 6.48	25.7 ± 3.89*	24.1 ± 2.55*^†^

Values are mean ± SD.

*R.D.* regional disease; *H.D.* homogeneous disease.

*p < 0.05 for compared with R.D. group.

^†^p < 0.05 for compared with H.D. group.

### Regional ECV vs. the whole regional ECV

**Figure 3:**
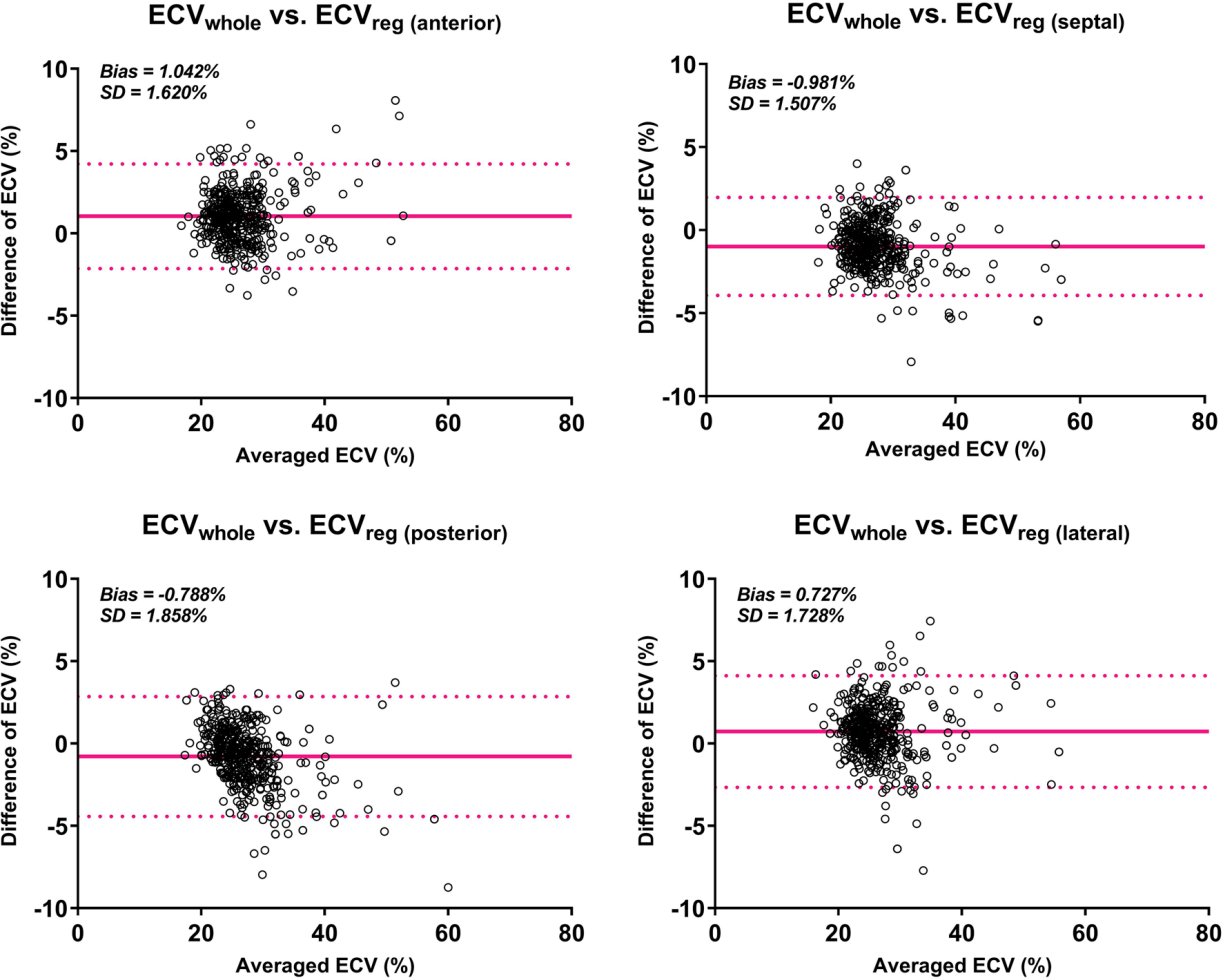
Bland–Altman plots of the mean differences between the whole regional ECV (ECV_whole_) and the regional ECV (ECV_reg_), which was derived from the anterior (upper left), septal (upper right), posterior (lower left) and lateral regions (lower right). The solid red horizontal line plots the mean difference, and the dashed red lines indicate the limits of agreement (differences from the mean of 1.96 SDs) for each region.

**Figure 4:**
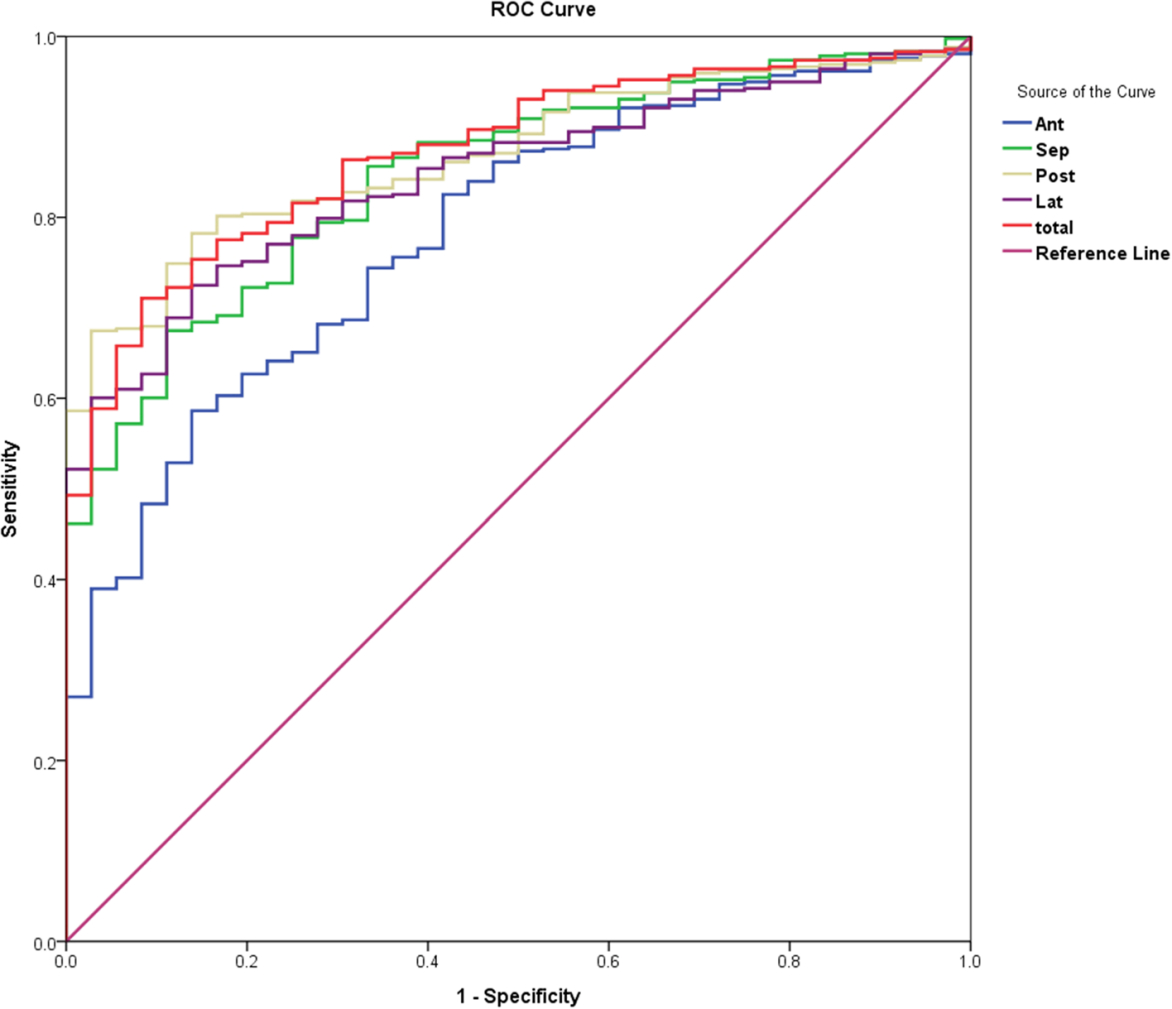
Receiver operating characteristic (ROC) curves for ECV measurements from each myocardial region and the whole region for detecting myocardial abnormalities.

Strong agreements between ECV_whole_ and ECV_reg_ were seen when measured from the anterior (ICC = 0.932, p < 0.001), septal (ICC = 0.948, p < 0.001), posterior (ICC = 0.938, p < 0.001) and lateral regions (ICC = 0.939, p < 0.001). Bland–Altman plots showing the mean difference between these two ECVs are shown in Fig. 3. The diagnostic power for detecting the myocardial abnormalities was similar between ECV_whole_ (AUC = 0.872, 95% CI [0.828, 0.917], p < 0.001) and ECV_reg_ for the septal (AUC = 0.847, 95% CI [0.795, 0.899], p < 0.001), posterior (AUC = 0.870, 95% CI [0.829, 0.910], p < 0.001), and lateral regions (AUC = 0.846, 95% CI [0.799, 0.892], p < 0.001) (Fig. 4). The ECV_reg_ measured in the anterior region showed significantly lower diagnostic performance (AUC = 0.786, 95% CI [0.718, 0.853], p < 0.001) than in the other regions (compared with septal, p = 0.0164; posterior, p = 0.0049; and lateral, p = 0.0166). The cut-off values for each myocardial region are listed in Table 3. The standard deviation among these cut-off values was 0.82%.

**Table 3:** The extracellular volume fraction (ECV) cut-off values determined from the sensitivity and specificity of each myocardial region.

	Cut-off value (%)	Sensitivity (%)	Specificity (%)
Anterior	23.7	68.2	72.2
Septal	24.8	79.4	72.2
Posterior	23.9	80.4	80.6
Lateral	22.8	79.9	72.2

### Interregional variation of ECV and its relationship with regional wall thickness

**Figure 5:**
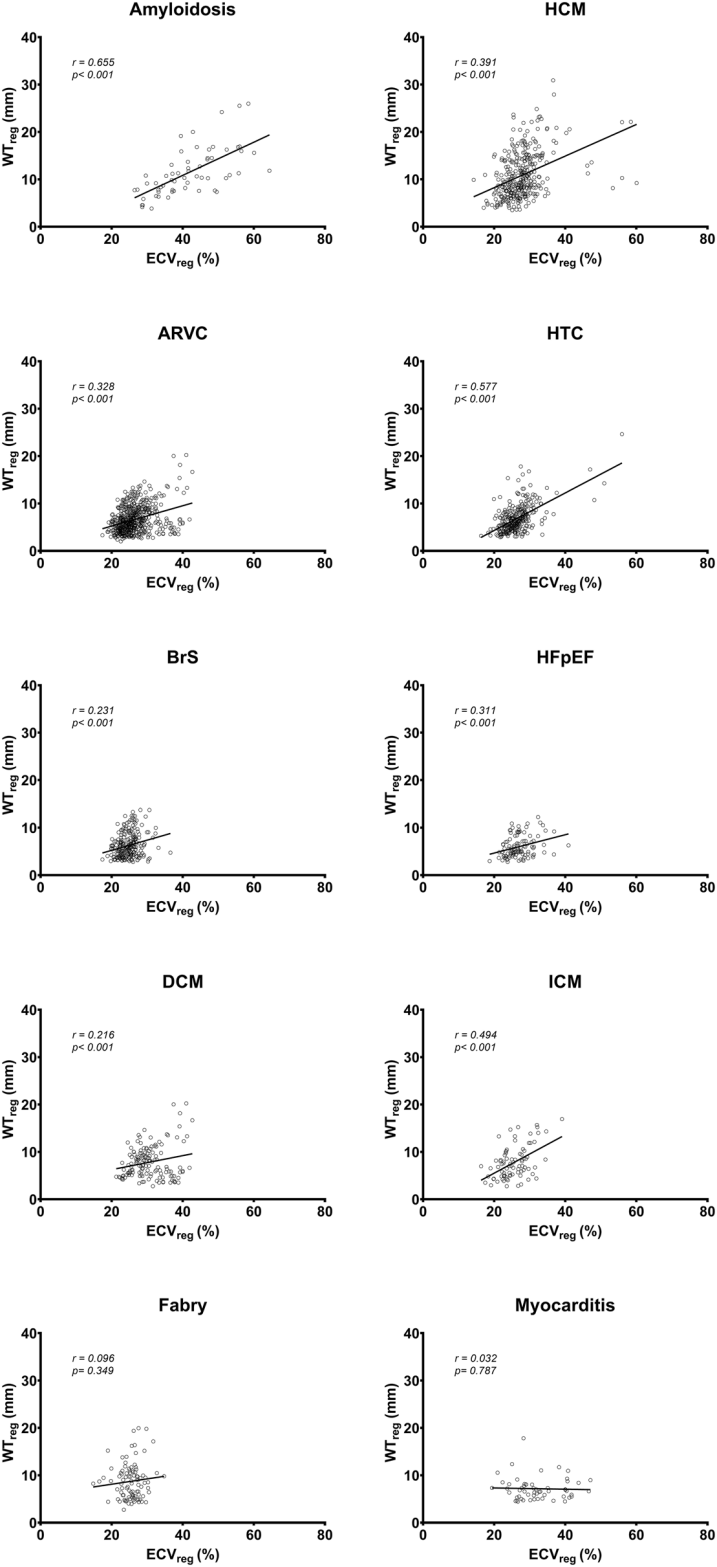
Linear regression between the regional ECV (ECV_reg_) and the corresponding regional wall thickness (WT_reg_) in the patient cohort. *ARVC* arrhythmogenic right ventricular cardiomyopathy; *BrS* Brugada syndrome; DCM, dilated cardiomyopathy; *Fabry* Fabry disease; *HCM* hypertrophic cardiomyopathy; *HTC* hypertensive cardiac disease; *HFpEF* heart failure with preserved ejection fraction; ICM.

**Figure 6:**
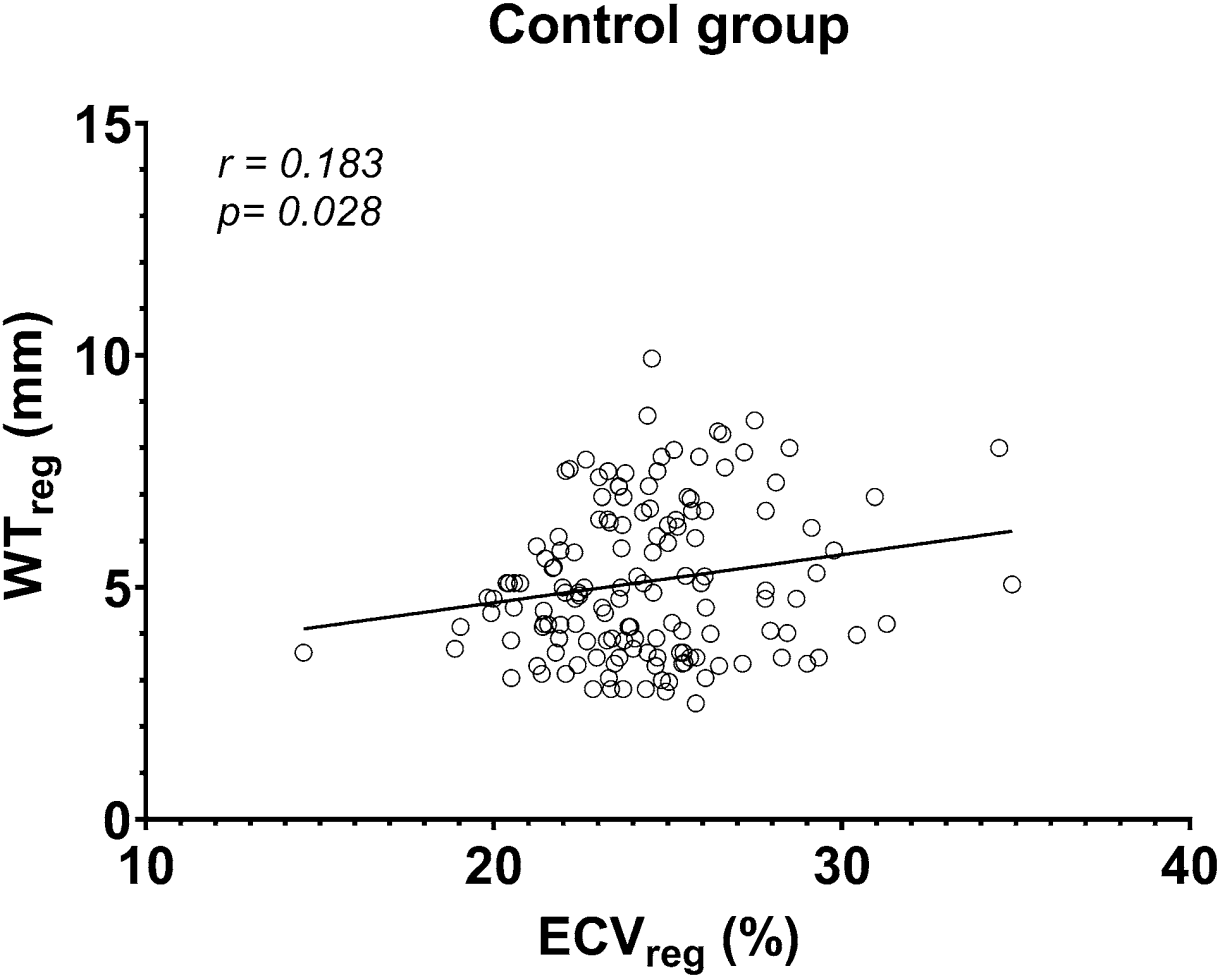
Linear regression between the regional ECV (ECV_reg_) and the corresponding regional wall thickness (WT_reg_) in the control group.

The inter*-*ECV within four different regions was significantly higher in the patient group than in the control group (compared with R.D. group: 2.13 ± 1.16% vs. 1.29 ± 0.52%, p < 0.001; compared with H.D. group: 1.82 ± 0.93% vs. 1.29 ± 0.52%, p < 0.001). In addition, the inter*-*ECV measured in the R.D. group was significantly higher than that measured in the H.D. group (2.13 ± 1.16% vs. 1.82 ± 0.93%, p = 0.016) (Fig. 1b). A relatively broad range of inter*-*ECV was found in the R.D. group compared with the H.D. (range 0.26–6.68% vs. 0.09–5.54%) and control groups (range 0.26–6.68% vs. range 0.38–2.77%). In addition, Fig. 5 showed that the ECV_reg_ was significantly correlated with the corresponding regional wall thickness in the patient cohort, except for patients with Fabry disease and myocarditis (Table 4). This significant correlation was also found in the control groups (r = 0.183; p = 0.028) (Fig. 6).

**Table 4:** The correlation coefficients between the regional ECV and the corresponding regional wall thickness in the patient cohort.

Patient group	Diagnosis	r	p value
H.D. group	Amyloidosis	0.655	< 0.001*
	ARVC	0.328	< 0.001*
	BrS	0.231	< 0.001*
	DCM	0.216	0.008*
	Fabry	0.096	0.349
R.D. group	HTC	0.577	< 0.001*
	HCM	0.391	< 0.001*
	HFpEF	0.311	< 0.001*
	ICM	0.494	< 0.001*
	Myocarditis	0.032	0.787

*H.D.* homogeneous disease; *R.D.* regional disease; *ARVC* arrhythmogenic right ventricular cardiomyopathy; *BrS* Brugada syndrome; *DCM* dilated cardiomyopathy; *Fabry* Fabry disease; *HTC* hypertensive cardiac disease; *HCM* hypertrophic cardiomyopathy; *HFpEF* heart failure with preserved ejection fraction; *ICM* ischemic cardiomyopathy.

*Statistical significance.

### ECV measurements from ROIs of different sizes

Table 5 compares the ECV results derived from ROIs of different sizes in the patient and control groups. No significant difference was found in the ECV measured from different sized ROIs for each myocardial region in either the patient or control group.

**Table 5:** Extracellular volume fraction (ECV) measurements of 5 different sized regions of interest (ROIs) drawn within four myocardial regions in the patient and control groups.

		Size 1	Size 2	Size 3	Size 4	Size 5	*p*-value
ROI group	Pixels	9	18	27	36	45	< 0.001
R.D. (n = 30)	Ant	27.8 ± 7.1	27.7 ± 6.6	27.6 ± 6.5	27.5 ± 5.4	27.3 ± 5.3	0.808
	Sep	29.5 ± 8.6	29.5 ± 7.5	29.5 ± 7.4	29.4 ± 7.1	29.4 ± 7.0	0.876
	Post	29.3 ± 8.5	29.3 ± 8.2	29.2 ± 8.1	29.1 ± 7.4	29.1 ± 6.9	0.734
	Lat	27.7 ± 7.7	27.6 ± 6.4	27.4 ± 6.3	27.4 ± 6.1	27.3 ± 6.0	0.793
H.D. (n = 30)	Ant	25.7 ± 4.8	25.7 ± 4.6	25.6 ± 4.5	25.5 ± 4.4	25.5 ± 4.3	0.820
	Sep	27.5 ± 4.6	27.5 ± 4.5	27.4 ± 4.4	27.4 ± 4.1	27.4 ± 4.0	0.886
	Post	27.8 ± 5.5	27.7 ± 5.2	27.6 ± 5.1	27.5 ± 4.9	27.5 ± 4.8	0.749
	Lat	26.2 ± 5.7	26.2 ± 5.4	26.1 ± 5.3	26.1 ± 4.1	26.1 ± 4.0	0.809
Ctr. (n = 30)	Ant	23.6 ± 2.8	23.5 ± 2.6	23.5 ± 2.5	23.5 ± 2.4	23.5 ± 2.3	0.848
	Sep	25.5 ± 2.6	25.5 ± 2.5	25.4 ± 2.4	25.4 ± 2.1	25.4 ± 2.0	0.896
	Post	24.8 ± 3.5	24.7 ± 3.2	24.6 ± 3.1	24.5 ± 3.0	24.5 ± 2.9	0.764
	Lat	24.2 ± 2.7	24.2 ± 2.4	24.1 ± 2.3	24.1 ± 2.1	24.1 ± 2.0	0.813

Values are mean ± SD.

*R.D.* regional disease; *H.D.* homogeneous disease; *CTR* control; *Ant.* anterior; *Sep* septal; *Post.* posterior; *Lat.* lateral.

### Reproducibility

**Figure 7:**
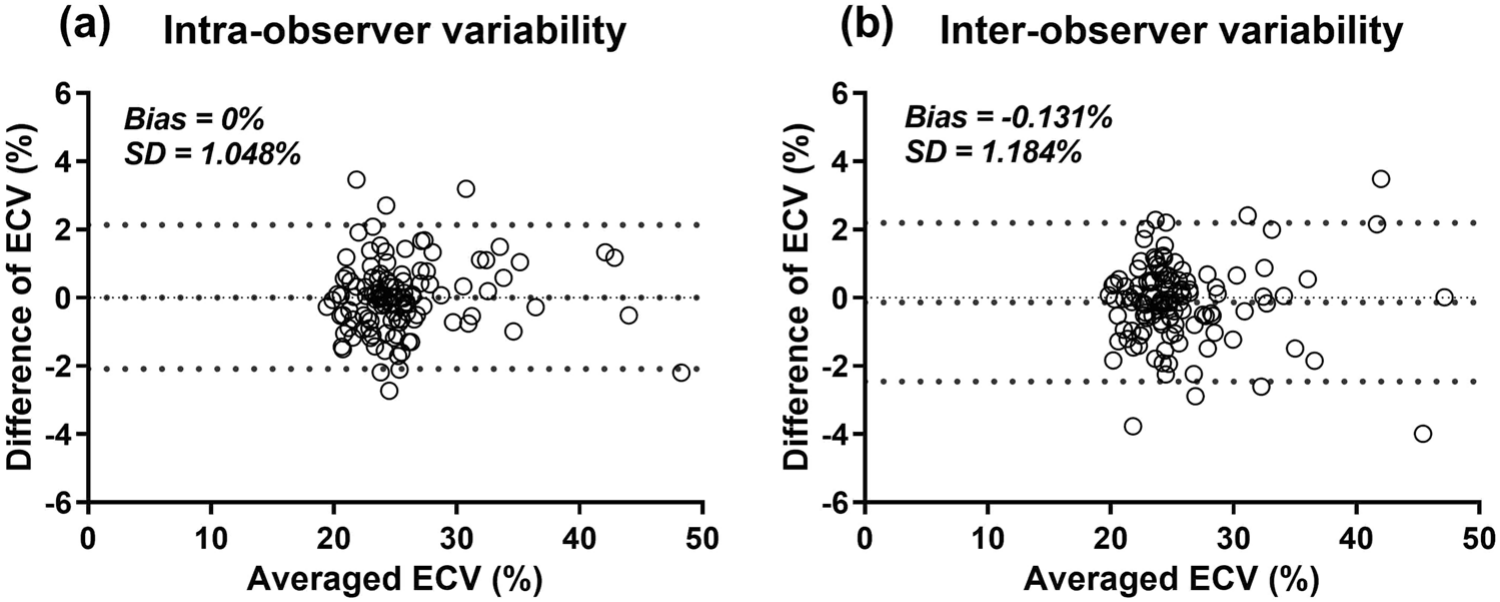
Intra-observer (**a**) and inter-observer (**b**) variabilities of regional ECV measurements from 120 myocardial regions in 30 subjects randomly selected from each group.

For the intra-observer variability, the ECV of repeated measurements range from 19.3% to 49.4% with medium 24.8%. The bias was 0% with 1.048% standard deviation of the absolute difference (Fig. 7a). For the inter-observer variability, the ECV of repeated measurements range from 19.3% to 49.4% with medium 24.5%. The bias was -0.131% with 1.184% standard deviation of the absolute difference (Fig. 7b).

## Discussion

In this study, we demonstrated that CMR-derived ECVs varied with the myocardial region and that this variation was significantly increased in patients with ventricular hypertrophy. Our results also showed that the diagnostic power of regional ECVs was similar to that of ECVs derived from the whole region except for the anterior region. These findings suggest that ECV measurement using the ROI-based method is feasible to detect myocardial abnormalities compared with the measurement from the whole region. However, regional ECV may lead to clinically significant errors if ECV was assessed and compared in different myocardial regions. In contrast, no significant difference was found in the ECVs from the 5 different ROI sizes. This finding suggests that the size of the ROI has less of an effect on the ECV measurement based on the ROI-based method.

CMR-derived ECV is a useful biomarker to quantify diffuse interstitial matrix ^2,5,8,12^ and predict adverse cardiovascular outcomes ^13–15^. ECV is most often measured using an ROI-based method, which assumes that the myocardial abnormality is homogeneously distributed (uniform ECV) within the noninfarcted myocardial regions. Kawel et al.^16^ used CMR to evaluate acquisition factors that may result in the variation of measured ECV, including magnetic field strength, cardiac phase and myocardial region in normal controls. They found that ECV did not vary significantly with field strength but did vary with cardiac phase and myocardial region. They concluded that ECV was significantly higher for the septum compared to the nonseptal myocardium in normal controls. Recently, Vita et al. demonstrated that ECV varies according to the myocardial region in patients with non-ischemic dilated cardiomyopathy ^17^. In this study, we further demonstrated that this interregional variation of ECV was more significant in the patient group than the control group and was positively associated with the corresponding regional wall thickness. These findings suggest that the degree of regional variation in ECV might be associated with the severity of ventricular hypertrophy.

Several segmentation approaches are used to quantify the ECV in clinical settings, including the entire LV myocardium, the septal myocardium and ROI in the noninfarcted myocardial region ^8^. Since CMR-derived ECV values may vary with myocardial region, it is essential to evaluate whether the position of the ROI affects the ability to differentiate myocardial abnormalities. In this study, we used ROC analysis to assess the diagnostic power of ECV measurements from each region and from the whole region of myocardium. Our results showed that the diagnostic power using regional ECV measured from the septal, posterior and lateral regions was similar to that measured from the whole region. This finding suggested that the ECV measurements from these regions can feasibly differentiate the myocardial abnormalities compared with the measurements from the whole myocardium. Our findings also indicated that ECV measurements from the anterior region had lower distinguishing power than the other regions. In this cohort, the SD was 0.82% for the ECV abnormality cut-off values in each myocardial region. For the intra- and interobserver variability tests, the bias SDs were 1.048% and 1.184%, respectively. Therefore, regional ECV variations did not lead to reclassification of the myocardial region being normal or abnormal, and no need to define the various cut-off values depending on myocardial regions.

CMR-derived ECV can be evaluated using the ROI-based method either from native and postcontrast T1 maps or directly from ECV mapping ^6^. In this study, we demonstrated that CMR-derived ECV measurements had significant interregional variation in both the control and patient groups. These variations were positively associated with the regional wall thickness. Therefore, caution must be exercised in estimating the ECV in patients with significant ventricular hypertrophy. The position of the ROI should be obtained in the same region or should cover the entire LV myocardium. In addition, our results showed that there was no significant difference in the ECVs from these five different sized ROIs. Nonetheless, we noted that the SD of the ECV within the ROI was lower in larger ROIs than in smaller ROIs. This finding may imply that one should select as large an ROI as possible without including ventricular blood and papillary muscle.

There are several limitations to this study. First, our study was done with a single T1 pulse sequence (MOLLI). Different sequences of T1 mapping have been reported to yield different absolute ECV values ^18^. The inter-ECV results were based on the relative difference in ECV among the four regions. Whether this variation is identical in different pulse sequences is unknown and needs further investigation. Second, our results indicated that the control group had two regional variations in ECV compared with four in the patient group (Fig. 2). The control group was smaller than the patient group, which may have limited our ability to detect subtle differences between regions. Whether the results of this study were merely a matter of sample size remains unknown. Nonetheless, there appears to be the same tendency of septal ECV being larger than the other regional ECVs in all groups. Third, although all subjects were carefully controlled in data acquisition and analysis, our results do not have histological evidence to support the suggestion that this interregional variation of ECV reflects a pathophysiological difference rather than a technical difference. Forth, regional variation of ECV was only performed in one representative mid-ventricular slice, future studies should consider apical and basal slices for more comprehensive assessments.

In conclusion, our study demonstrated that CMR-derived ECVs vary with myocardial region in both the patient and control groups. This interregional variation is associated with the severity of ventricular hypertrophy. Regional ECV is feasible for differentiating myocardial abnormalities compared with ECV derived from the whole region except for in the anterior region. When conducting ECV measurements using the ROI-based method, the position of the ROI is essential for comparing ROIs within the same region, but the measured size of the ROI is less critical to consistencies in ECV measurement.

## Materials and methods

### Ethics statement

The research was approved by the institutional review board of the National Taiwan University Hospital Ethics Committee. The study was conducted in accordance with the approved guidelines. All study participants provided written informed consent.

### Study population

Between January 2017 and October 2019, 647 subjects undergoing clinical or research CMR were retrospectively recruited for T1 mapping with the calculation of ECV. Exclusion criteria included subjects with suboptimal image quality due to arrhythmia (n = 34), examination without intravenous contrast administration (n = 22), and LGE images with hyperenhancement (n = 137). The final cohort consisted of 418 patients and 36 normal controls. Patients were divided into two groups, the regional disease group (R.D. group) and the homogeneous disease group (H.D. group) depending on if the distribution of cardiomyopathy was localized or infiltrative. Clinical and demographic information, including underlying cardiac diagnosis, was collected.

### Imaging acquisition

CMR was performed on a 1.5-T Magnetom Aera (Siemens Healthcare, Erlangen, Germany) with a 30-channel cardiac coil array. Myocardial T1 mapping was performed with an electrocardiography (ECG)-triggered modified Look-Locker inversion recovery (MOLLI) pulse sequence before and 10 min after 0.15 mmol/kg intravenous administration of the gadolinium-based contrast agent (Dotarem, Guerbet, France). The MOLLI protocol used a 5(3)3 sampling scheme for native T1 mapping and a 4(1)3(1)2 sampling scheme for postcontrast T1 mapping. Scan parameters were as follows: TE/TR 1.14/2.7 ms; flip angle 35°; bandwidth 977 Hz/Px; minimum TI 125–150 ms; TI increment 80 ms; pixel-spacing 1.36 × 1.36 mm^2^; slice thickness 8 mm; iPAT factor (GRAPPA) 2. Cine MRI was performed using a segmented balanced steady-state gradient echo pulse sequence with a retrospective ECG R-wave trigger. Scan parameters were as follows: TE/TR 1.6/3.0 ms; flip angle 50–70°; bandwidth 975 Hz/Px; pixel-spacing 1.25 × 1.25 mm^2^; slice thickness 8 mm; gap 2 mm; iPAT factor (GRAPPA) 2. A total of 10–12 short axis slices were obtained, depending on cardiac size. Thirty cardiac phases were acquired for each level. After postcontrast T1 acquisition, LGE images were acquired using an ECG-triggered phase-sensitive inversion recovery prepared segmented fast gradient-echo pulse sequence to identify the focal fibrosis or scarring.

### Imaging analysis

Commercial postprocessing software (cvi42, Circle Cardiovascular imaging, Calgary, AB, Canada) was used to analyze the LV function, regional wall thickness and ECV offline. Maximum wall thickness was measured from end-diastolic short-axis cine image. The wall thickness of the anterior, septal, posterior and lateral segments was measured at the same level of the T1 mapping. The ECV was calculated from native and postcontrast T1 maps using a region-based method and was then calibrated with the last available hematocrit data. Four different regions, including the anterior, septal, posterior and lateral wall regions of the LV, and the area in the central area of the LV cavity were drawn on the T1 map at the mid-ventricular slice. The position of the ROI was carefully drawn to avoid the inclusion of trabeculation, blood or pericardium in the thin-walled parts of the myocardium. The regional ECVs (ECV_reg_) measured from four different regions and the whole regional ECV (ECV_whole_) calculated from the four ECV_reg_ were compared in each group and among the groups. In addition, the interregional variation of ECV (inter-ECV) was assessed by measuring the standard deviation (SD) of ECV_reg_ among these four regions. We hypothesized that the variations in ECV might be associated with the ventricular wall thickness. The ECV_reg_ for all subjects were correlated with the corresponding regional wall thickness. Five different sized ROIs drawn within the myocardial region were selected to quantify the ECV in 90 subjects who were randomly selected from each group. To standardize the analyzed procedures, five 3 × 3 ROI pixels were placed equally across the myocardial regions. Five different ECV sizes were measured from one to five ROI 3 × 3 pixels. To evaluate the reproducibility of the ECV measurements, intra- and interobserver variabilities were studied in 120 myocardial regions of 30 subjects randomly selected from each group.

### Statistical analysis

All continuous data were first tested for fitting the normal distribution using the Shapiro–Wilk test. Continuous variables were expressed as the means and SD, and categorical variables were expressed as the percentages. Since the Shapiro–Wilk test showed that most ECV values were not normally distributed, all statistical analyses were performed using nonparametric methods. Group comparisons between the measured ECV in each region were analyzed with the Mann–Whitney U test. Regional comparisons among the measured ECVs for each group were performed by using the Kruskal–Wallis test, and the Mann–Whitney U test was used for post-hoc analysis. The statistical tests were two-tailed, and statistical significance was defined as P < 0.05. Spearman’s rank correlation was used to obtain correlation coefficients between continuous variables of interest. Agreements between the measurements were assessed via the intraclass correlation (ICC) coefficient uing a two-way random-effects model. The receiver operating characteristic (ROC) analysis was performed to evaluate the diagnostic power to differentiate the patient and control group for each regional ECV and the whole regional ECV. The data were analyzed using SPSS (version 20, Statistical Package for the Social Sciences, International Business Machines, Inc., Armonk, New York, USA) and Prism (version 5.01, GraphPad Software, Inc., La Jolla, California, USA).
