# Author Correction: CMR-derived ECVs vary with myocardial region and associate with the regional wall thickness

**DOI:** 10.1038/s41598-021-87655-4

**Published:** 2021-04-09

**Authors:** Mao-Yuan Su, Kuei-Yuan Hou, Ming-Hung Liu, Tien-Min Lin, Jyh-Ming Jimmy Juang, Lian-Yu Lin, Cho-Kai Wu, Hsi-Yu Yu, Shun-Chung Yang, Yu-Sen Huang, Emi Niisato, Yeun-Chung Chang

**Affiliations:** 1 Department of Medical Imaging, National Taiwan University Hospital, Taipei, 10002 Taiwan, ROC; 2 Department of Medical Imaging, Cathay General Hospital, Taipei, 10630 Taiwan, ROC; 3 Department of Biomedical Imaging and Radiological Sciences, National Yang-Ming University, Taipei, 11221 Taiwan, ROC; 4 Cardiovascular Center and Division of Cardiology, Department of Internal Medicine, National Taiwan University Hospital, Taipei, 10002 Taiwan, ROC; 5 Department of Surgery, National Taiwan University Hospital, Taipei, 10002 Taiwan; 6 Siemens Healthcare Limited, Taipei, 11503 Taiwan, ROC; 7 Department of Medical Imaging and Radiological Technology, Yuanpei University of Medical Technology, Hsinchu, Taiwan, ROC

Correction to: *Scientific Reports* 10.1038/s41598-020-78043-5, published online 01 December 2020

In the original version of this Article, Shun-Chung Yang and Yu-Sen Huang were incorrectly affiliated with ‘Cardiovascular Center and Division of Cardiology, Department of Internal Medicine, National Taiwan University Hospital, Taipei 10002, Taiwan, ROC.’ The correct affiliation is listed below.

Department of Medical Imaging, National Taiwan University Hospital, Taipei 10002, Taiwan, ROC.

Furthermore, the affiliation ‘Department of Medical Imaging, National Taiwan University Hospital, No.7, Chung‑Shan South Road, Taipei 100, Taiwan, ROC.’ was a duplicate of affiliation ‘Department of Medical Imaging, National Taiwan University Hospital, Taipei 10002, Taiwan, ROC.’. The duplicate has been removed for Yeun-Chung Chang.

These errors have now been corrected in the PDF and HTML versions of the Article.

